# Toward Normalizing Inclusive Design by Uncovering Patient Experiences of a Web Portal in a Dental Hospital: Mixed Methods Study

**DOI:** 10.2196/74275

**Published:** 2025-08-14

**Authors:** Adeola Bamgboje-Ayodele, Jacques Raubenheimer, Anna Paonne, Jessica Yi, Tamasha Jayawardena, Shilpi Ajwani, Eugenia Szuba, Tanmay Chowdhury, Phu Dao, Mostafa Ahmed, Amy Von Huben, Martin Howell, Nicole Nixon, Adam Dunn, Aaron Jones, Melissa Baysari

**Affiliations:** 1 Digital Health Human Factors Research Group, Sydney Nursing School, Faculty of Medicine and Health, The University of Sydney, Camperdown, Australia; 2 Discipline of Design, School of Architecture, Design and Planning, The University of Sydney, Room 585, Wilkinson Building (G04), 148 City Road, Darlington, NSW, 2008, Australia, 61 2 9351 9644; 3 Sydney Pharmacy School, Faculty of Medicine and Health, The University of Sydney, Camperdown, Australia; 4 Sydney Local Health District, New South Wales, Australia; 5 Five Faces, Queensland, Australia; 6 Biomedical Informatics and Digital Health, Faculty of Medicine and Health, The University of Sydney, Camperdown, Australia

**Keywords:** patient portal, inclusive design, usability, patient experience

## Abstract

**Background:**

Patient portals can improve care delivery and the efficient use of health care resources. Barriers to uptake can hinder the realization of expected benefits for health services and patients, particularly for older adults (≥65 y) and those who are culturally and linguistically diverse (CALD).

**Objective:**

This study aimed to evaluate the usability and overall patient experience of a patient portal, particularly for CALD and older adults.

**Methods:**

A mixed methods study at a dental hospital was performed including a patient experience survey that was administered before (2111 patients) and after (2445 patients) portal implementation to all patients, semistructured interviews (18 patients), and a usability survey (235 patients) that was administered to all patients who had registered to use the portal and had an appointment at the clinic. Also, we conducted a scenario-based usability evaluation (17 patients) with CALD and people.

**Results:**

The patient experience survey revealed that the portal had no impact on the ease of changing appointments when the clinic with the portal (Wilcoxon z=−1.62; *P*=.05) was compared with clinics without the portal in the same hospital (z=−1.54; *P*=.06). For the usability survey, >82% were completed by patients and 18% by carers, and 47.3% of respondents were older adults (≥65 years old). The majority spoke English (n=191, 81.3%), while others preferred to speak other languages (n=44, 18.7%) and were identified as CALD. Adult participants (18‐64 years old) reported that the portal was easy to use and simple. However, design problems, including inadequate incorporation of inclusivity, were found to more often limit usability for older (≥65) and CALD people. The overall Simplified System Usability Scale (SUS) mean score was 63.3 (95% CI 60.9‐65.7). Adult participants reflected higher SUS scores than older participants (*F*=10.4; *P*=.001). Interview results revealed how the portal was used and barriers to its uptake. Barriers related to poor usability and gaps in the implementation approach (eg, limited awareness of the purpose of the portal). Usability evaluations indicated that all participants could log in with the one-time password (OTP), but most were assisted. Only 3 of 17 participants were able to send a message to the clinic. Other usability problems identified were focused on display, content, layout, functionality, and navigation categories.

**Conclusions:**

The use of inclusive design principles when designing patient portals is necessary for successfully engaging all patients. Our study highlights the importance of normalizing inclusive designs in patient portals to ensure that priority populations, such as older and CALD people, are not marginalized by design. We provide recommendations to guide future design and implementation of patient portals.

## Introduction

Demand for medical specialist appointments in publicly funded health services continues to rise worldwide [1]. To cater to the growing demand and limited health resources, mechanisms to ensure efficiency in health service usage continue to remain a high priority for health services [2]. Failure to attend (FTA) a clinic appointment is one mechanism by which health service usage is measured, as it identifies outpatient clinic appointments that are not attended by patients with no warning to the health service [3]. Patients who forget an appointment or experience difficulties canceling appointments (eg, called but could not get through) are frequently cited reasons for failing to attend [4]. Evidence suggests that patient portals can reduce FTA rates if they are usable and used [5].

Patient portals are web-based applications that are managed by health care organizations that aim to support patient appointment bookings, reminders, and communication with health care providers via secure messaging from anywhere via the internet [6]. Patient portals are beneficial to health services as they have been reported to reduce missed appointments and improve efficiency [7]. For patients, portals have been reported to benefit clinician-patient relationships, health status awareness, and adherence to therapy and medications [8,9].

Despite the potential benefits that patient portals can provide, existing studies have reported several barriers to uptake and implementation, such as poor usability and lack of awareness [6,10-13]. Patient portals can also inadvertently result in widening inequalities in access to health care for people from culturally and linguistically diverse (CALD) backgrounds, and older adults, as they often face challenges when navigating digital systems and the health care system, like language barriers and lower digital literacy [14]. Specifically, older adults are more likely to experience visual impairment, memory decline, hearing loss, and other age-related cognitive changes [15], which may lead to technologies being unusable, preventing digital inclusion [16,17]. Usability and inclusivity are inextricably linked. Inclusivity is threatened when technology usability issues are disproportionately experienced by, or exacerbated in, specific groups (such as those with low digital literacy) [18]. A recent study prototyped, tested, and evaluated interactional features embedded in a patient portal with 503 participants in the United Arab Emirates to support inclusion of patients with or without vision or hearing impairments [19]. They found and recommended that accessible features should be included in the existing portal [19].

To improve usability and uptake, existing studies have recommended training to enhance patient digital literacy skills [17,20]. Digital literacy has been shown to be a social determinant of health in old age, which requires the need to learn skills to support the use of digital technologies [21]. However, rather than relying on patients to upskill (or receive training) to be able to use patient portals, these portals could be inclusively designed and easy to use. Failure to ensure inclusive design will continue to widen the gap between those able to use digital technologies and those unable to do so [22]. In a systematic review of 18 studies on interventions to increase patient portal use in vulnerable populations, they found that only one study reported on disparities in use, limiting our understanding of the gap between portal users and nonusers [23]. Limited research has evaluated the design of patient portals to determine if they are inclusive and usable in real-world settings, particularly for CALD and older people. The aims of this study were to (1) evaluate the impact of implementing a web portal on patient experience in a real-world setting and to (2) assess the usability of the web portal and make recommendations to optimize its design, particularly for CALD and older people.

## Methods

### Study Design

This was a mixed methods study that included a pre-post observational (patient experience survey) component, semistructured interviews, usability survey, and scenario-based usability evaluation. A mixed method approach was used to leverage the strengths of both quantitative and qualitative data and produce a more complete understanding of the phenomenon of interest. Quantitative methods provided insights into whether there were usability problems and for whom, while qualitative methods provided insights into what the problems were.

### Setting

The study was conducted at a publicly funded oral health service in New South Wales, Australia. The oral health service includes a tertiary dental hospital, a major teaching facility, and community-based (outpatient) oral health clinics across 4 locations. It provides general and specialist oral health services to children and eligible adult patients. Eligible adults are those who qualify for government subsidies for health care specifically aimed at individuals and families who are socially and economically disadvantaged. The oral health service has 160 dental chairs and sees, on average, 12,000 patients a month. The facility is operated by administrative staff and dental clinicians, including registered dentists, specialists, oral health therapists, postgraduate students, dental technicians, nurses, and allied health. This study evaluated the implementation of a patient portal at a single clinic within the oral health service.

### Patient Portal Description

This study focused on a web-based application for managing patient appointments in outpatient clinics known as Florence v1.0, which went live at the clinic on December 6, 2023. The web application allows patients to view upcoming appointments, send messages to or receive messages from clinics, confirm upcoming appointments, request cancellation, or request a reschedule of their appointments. Florence was only able to be delivered in English when this study was conducted.

### Participant Recruitment and Data Collection Procedure

#### Patient Experience Survey

Routinely administered patient-reported experience measure (PREM) surveys are collected at the dental hospital. For this study, an additional item relevant to Florence was included and sent to all patients across all the clinics of the dental hospital before and after Florence was implemented at the single clinic. The additional item was sent to each patient one day after their appointment and was stated as, “it was easy for me to change my appointment when I needed to.” We collected and analyzed PREM survey data for all patients who attended all clinics of the dental hospital (including the clinic where Florence was implemented) for a 3-month period before Florence was implemented (September 6-December 5, 2023). After a 3-month period to resolve technical and staffing issues associated with implementation, postimplementation data were collected during the subsequent 3-month period (March 6-June 5, 2024). Data from the other clinics were used as a control against which to compare the PREM results from the Florence clinic.

#### Usability Survey

An online postimplementation usability survey was conducted. The link to the survey was sent to all eligible patients by a Florence text message. Eligible participants were patients aged 18 years and older who could provide informed consent, received care at the clinic where Florence was used, had registered to use Florence, and had an appointment at the clinic following commencement of Florence. The survey included questions to collect demographic information from patients. It also included the validated 10-item Simplified System Usability Scale (SUS), which has been adapted for adults with cognitive impairment and old age [24]. This version of the SUS survey was used for all participants. At the end of the survey, patients were asked whether they would like to participate in a follow-up interview and a usability test.

#### Interviews and Usability Testing

Semistructured interviews and scenario-based usability testing were conducted with eligible participants (Can be found in Appendix A in Multimedia Appendix 1 for interview guide and Appendix B in Multimedia Appendix 1 for usability test scenarios). Eligible participants for the interviews were patients aged 18 years and older who could provide informed consent and were receiving care where and when Florence was used. Participants were initially recruited through the usability survey. Interested participants were contacted by email to schedule the interview or usability test. We also recruited CALD participants for the interviews or usability tests in person at the clinic. To participate in the usability test, patients needed to be CALD aged 65 years and older, or both (ie, in accordance with the definition for older participants in this study). The usability tests focused on the 6 non-English languages most frequently spoken by patients attending the clinic: Cantonese, Mandarin, Greek, Arabic, Vietnamese, and Italian. An interpreter or a multicultural support worker, who spoke one of the 6 languages, was available at the clinic to help recruit culturally diverse and linguistically diverse participants who had registered to use Florence and had an appointment at the clinic. All participants were observed performing 7 tasks (eg, logging in with a one-time password [OTP], see Appendix A in Multimedia Appendix 1) by ABA, and either JY or TJ. Participants who took part in the usability test or interviews were given an Aus $20 gift voucher (ie, US $13). Although existing research suggests that 12‐15 interviews are generally adequate for qualitative research [25,26], data collection ended when no new information emerged, and thematic saturation was achieved. The interviews and verbalizations during the usability tests were audio-recorded and transcribed for analysis. Handwritten field notes of the challenges that participants encountered in the usability tests were also taken. As usability tests are observational in nature, they complemented the semistructured interviews, thus providing richer insights.

### Data Analysis

For the PREM data, we compared the clinic that implemented Florence to other clinics at the dental hospital. We performed an ordered logistic regression to evaluate the degree to which respondents felt it was easy for them to change their appointments (5 rating levels: strongly agree [1], agree [2], undecided [3], disagree [4], and strongly disagree [5]). The covariates were age, pre- versus postimplementation of Florence, and clinics with Florence versus clinics without Florence in the same dental hospital.

The usability survey and scenario-based usability evaluation data were analyzed descriptively. In line with previous research [27], SUS scores for each question were summed and then multiplied by 2.5 to convert the original scores ranging from 0 to 40 to a range of 0 to 100. SUS scores above 68 indicate average usability and a score below 68 is considered to be below average [28]. As the age distribution (Table 1) was predominantly (>90%) individuals in the 2 age brackets, 35‐64 years and 65‐84 years, we collapsed age into 2 groups, namely adults (18–64) and older adults (≥65). A general linear model ANOVA was used to test whether CALD status, age (as a binary, with 18‐64 compared to ≥65), or the interaction between CALD status and age predicted the SUS scores. Two Human Factors researchers reviewed each usability problem identified during usability testing and those that emerged during interviews and developed a set of design recommendations. These design recommendations were validated with the IT staff, including a digital product analyst and the designers of the portal, in two 30-minute meetings.

**Table 1.** Patient-reported experience measures survey completions.

Variables	Preimplementation measures, n (%)	Postimplementation measures, n (%)	Standardized difference
Patients	2116 (46.3)	2455 (53.6)	
Status			0.07
Carer	132 (6.2)	166 (6.7)	
Parent	242 (11.4)	254 (10.3)	
Patient	1742 (82.3)	2035 (82.8)	
Age (years)			0.10
18‐34	164 (7.7)	158 (6.4)	
35‐64	1003 (47.4)	1093 (44.5)	
65‐84	898 (42.4)	1122 (45.7)	
85+	51 (2.4)	82 (3.3)	
Clinic			−0.04
Florence clinic	578 (27.3)	713 (29.0)	
Non-Florence (or other) clinics	1538 (72.6)	1742 (70.9)	

Deidentified transcripts and field notes from usability testing were thematically analyzed independently by ABA, who first identified and coded the transcribed data through inductive thematic analysis, using a data-driven approach to understand participants’ experiences and perceptions [29,30]. Our analytical approach included data familiarization, coding, generating initial themes, reviewing potential themes, defining and naming themes, and producing the report [31]. Codes were later reviewed by MB. Disagreements in themes were discussed in meetings to clarify the rationale and how a theme emerged until a consensus was reached. Findings from the usability tests were later mapped to an existing guideline for designing for older adults [16].

Data from the usability survey provided insights into whether there were usability problems and for whom. Data integration first occurred at this point, as data from the usability survey confirmed the need to focus on people for subsequent interviews and usability tests. Data from the interviews confirmed the demographic of interest and provided in-depth insights into what the problems were within and beyond the portal, while data from the usability test focused on the demographic of interest and provided richer insights into the portal’s interface. Findings from the survey, interview, and usability test were integrated to form a rich description of patients’ experiences.

### Ethical Considerations

Ethics approval was obtained from the hospital’s Human Research Ethics Committee (X23-0274 & 2023/ETH01612). Consent was obtained from all participants, who were informed of their right to withdraw from the study at any time. All identifying characteristics have been removed to protect anonymity.

## Results

### Patient Experience (Pre-Post) Survey

In total, 4571 respondents completed the PREMs surveys in the designated study period. Descriptive statistics for the sample are shown in Table 1. A total of 2116 surveys were completed in the preimplementation period (Sept 6 to Dec 5, 2023) and 2455 in the postimplementation period (Mar 6 to Jun 5, 2024).

**Figure 1.**
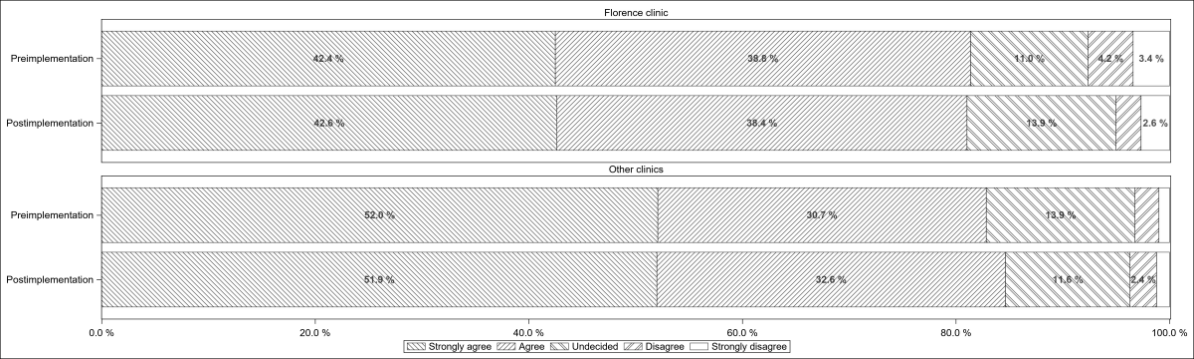
Ease of changing appointments before versus post implementation.

When asked how easy it was for participants to change their appointment when they needed to, fewer participants agreed that this task was easy at the Florence clinic compared with the non-Florence (or other) clinics (Figure 1). However, there was no significant change from preimplementation to postimplementation for either the Florence clinic (Wilcoxon z=−1.62, *P*=.052) or non-Florence clinics (z=−1.54, *P*=.06). Patients aged 65 years and older were slightly less likely to indicate that changing appointments was easy, compared with younger patients (odds ratio 1.13, 95% CI 1.00‐1.30).

### Usability Experience Survey

In addition to the routine PREMs survey, another online survey was disseminated by text message to the mobile phone numbers of all 2309 patients who were eligible. Initially, 442 patients followed the link, although 104 did not consent to complete the survey. A further 103 respondents consented but did not complete enough of the survey items to provide usable data, leaving a final sample of 235 respondents (10% of those invited).

Most of the respondents were patients (n=219, 93.2%), with 13 carers (5.5%) and 3 parents (1.3%). There were more males (n=124, 52.8%) than females (n=107, 45.5%), and a minority were nonbinary (n=4, 1.7%). The majority of the respondents spoke English (n=191, 81.3%), while others preferred to speak other languages (n=44, 18.7%) and were identified as CALD. Of these, Chinese or Mandarin was the most frequently preferred language (n=21, 50%). A total of 127 (54%) respondents were less than 65 years, and 108 (46%) were 65 years and above.

**Figure 2.**
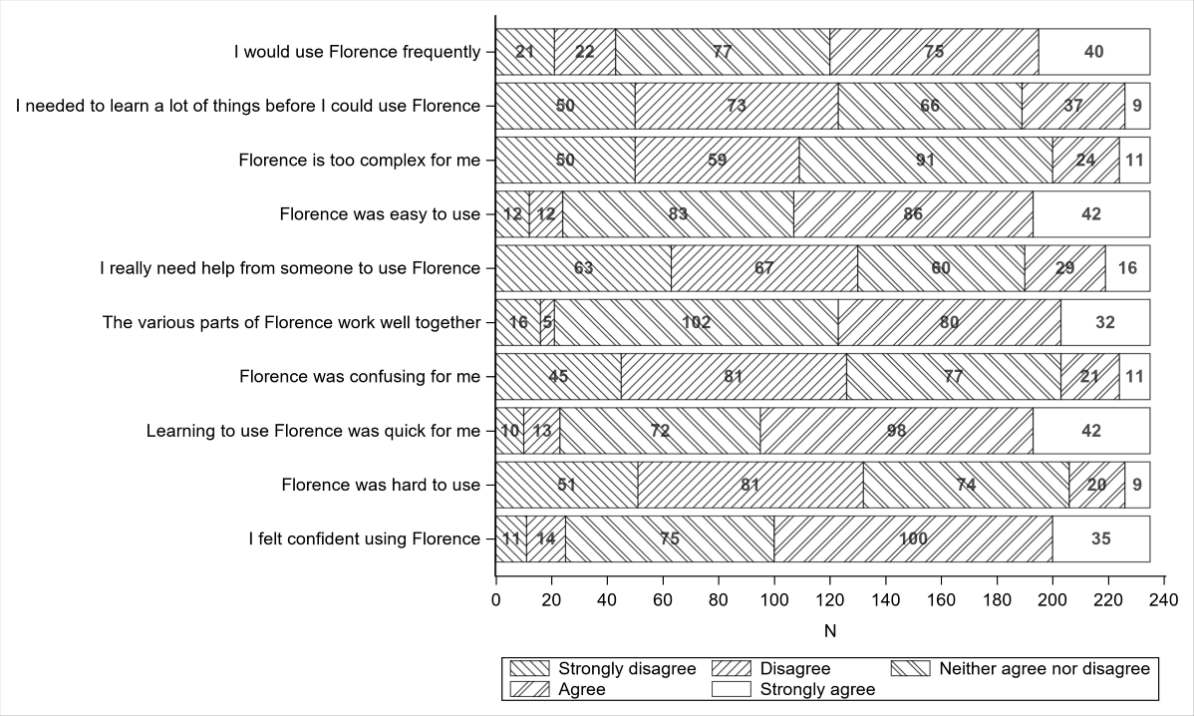
Simplified System Usability Scale responses.

The participants’ overall mean SUS score was 63.3 (95% CI 60.9‐65.7). Unadjusted SUS responses for all participants are aggregated in Figure 2, revealing moderately high scores for ease of use (3.57), ease of learning (3.63), and confidence using the portal (3.57).

The ANOVA results showed that age significantly predicted SUS scores (*F*=10.4; *P*=.001), with younger participants reporting the portal to be more usable than older participants (Table 2). CALD status did not predict SUS scores, and the interaction between age and CALD status was also not statistically significant.

**Table 2.** Age distribution of respondents and Simplified System Usability Scale scores across older and younger participants.

Age category and group (years)	Mean SUS scores (95% CI)	Participants, n (%)
65 years and younger	68.8 (63.55‐70.07)	
18‐34		8 (3.4)
35‐64		119 (50.6)
65 years and older	59.1 (55.89‐62.49)	
65‐84		103 (43.8)
85+		5 (2.1)

### Semistructured Interviews

In total, 18 patients participated in semistructured interviews. There were more females (n=10, 56%) than males (n=8, 44%) and the mean age of participants was 56 (range 27‐75) years. With respect to devices used at home, half of the participants reported using a combination of smartphone, tablet, and computer, 4 (22%) reported using 2 of those devices, and 5 (28%) reported using one device in their daily activities. The majority (n=16, 89%) of the participants reported that they use these devices regularly, but 2 stated that they ask friends or relatives to help them. While all participants spoke English, some (n=4) reported a preference for other languages including Mandarin, Cantonese, Arabic, and Filipino.

Interviews ran for an average of 15 minutes. The findings were grouped into 4 themes: uses of the portal, positive views on the portal, barriers to portal uptake, and recommendations for improving the portal.

### How the Portal Was Used

How the portal was used emerged as a key theme from the interviews. Most participants explained that they used the portal to organize and keep track of their appointment dates and times. The portal was seen as an appointment diary that made participants feel like they were in control of their appointments:

*I felt like that I was in control. I was able to manage my appointments and organise my appointment, my day in such a way that I that I was able to make the appointment.*

[P005, female, 47 years]

In addition, the portal was reportedly helpful for cancelling appointments:

*I mean it gives you a chance to cancel your appointment up until the day before, I think, which can be advantageous in some circumstances.*

[P01]

Similarly, the portal was used to confirm patient appointments, to let the clinic know whether the patient would attend or not:

*You go in and basically it’s just you go in and you just confirm*.

[P07, female, 54 years]

Similarly, it was stated that the portal was used by participants to leave messages for the clinic. Participants indicated that they preferred using Florence to calling the clinic by phone.

*Yeah, I think better than I call. I call someone, don’t have any answer…When I call the hospital, I take long time to wait*

[P04, male, 65 years, Mandarin speaking]

### Positive Views on the Portal

There was positive feedback about the portal. Specifically, many participants reported that the portal was very easy to use, simple, and straightforward. For example, a patient said,

*if I can do it, anyone can do it…because it’s easy to read and it asks you simple, straightforward questions*.

[P005, female, 47 years]

Others reported that the layout was reasonable, logical, and easy to learn as the instructions could be followed in a step-wise fashion.

*I think the arrangement was quite, like, reasonable or logical. So you just follow what’s going next. You just follow them step by step*.

[P018, female, 70 years, Cantonese speaking]

In addition, some participants mentioned that the portal was easy to access, particularly because there was no need to remember or input any password:

*you only have to put your mobile number in and it sends you the link and then you can go in so that’s really good. So you don’t have to put a password and all that, so I thought that was a really good feature*.

[P012, male, 43 years]

Another participant said,

*for someone like me to be able to access it without any problems, it must be pretty well designed*

[P03, male 68 years]

In addition to the positive feedback, one reported benefit of the portal was its ability to save time for patients. For example, when compared with the usual practice of making phone calls to the clinic, participants stated that the portal was preferred:

*I thought it was quite easy to use and great, rather than, you know, calling up and waiting. It was much easier*.

[P012, male, 43 years]

Another stated:

*I actually love it. It saves so much time*.

[P02, female, 56 years]

### Barriers to Portal Uptake

#### Poor Usability

Usability presented the main challenge with using the portal for some participants. Some participants explained that it was difficult to send messages to the clinic using the portal:

*to actually write a message that’s just advising them on what’s happening on my end, I couldn’t*.

[P001, male, 51 years.]

Also, a few participants stated that it was difficult to access the link to the portal on a subsequent occasion, after the initial login, as the text message that displays the link to the portal may be inadvertently deleted by the user. For example, a participant said,

“*if I delete the text message that I receive, that says, click on to Florence, I then don’t know where to find this information*.”

[P007, female, 54 years]

It was further reported that it was challenging to copy and paste the OTP when attempting to access the portal via a smartphone, as this requires a certain level of digital literacy; otherwise, the user may experience a cycle of generating links and OTPs without being able to log in successfully.

*what happens is you click onto the link, and then it asks you to request a code. Now that code, then gets sent through via text message, but what happens is, if you don’t copy it, and go back to the where it’s where you’re supposed to… you kind of lose where to go back and copy it*.

[P007, female, 54 years]

Furthermore, many participants who were CALD and/or older people explained that the portal was neither inclusive nor user friendly. All CALD participants reported that the portal was only available in English language and that they were unable to read the instructions written in English. For example, one participant said, “I think, only the problem is language” (P004, male, 65 years, Mandarin speaking), and another said, “if it is Chinese, if I can read it, then it will be easier for me to use” (P018, female, 70 years, Cantonese speaking). For older participants, they mentioned that they did not understand the “tick” and “cross” buttons on the portal as the purpose of those buttons should have been stated in plain language (eg, yes or no), which would have been more understandable to them.

*I’m 72, I think … older people, like it took me a while looking at it to get oh, yeah, of course a tick, where if it was a yes or a no it would be much easier*.

[P010, male, 72 years]

Another older participant stated that,

*I also think it kind of, like complicated, well for me anyway. I’m older and so I didn’t grow up with phones and computers. So yeah I constantly find those sorts of things quite difficult, compared just actually someone picking up a phone and talking to you.*

[P009, male, 62 years]

#### Gaps in Implementation Approach

The implementation approach was deemed suboptimal as challenges emerged around a lack of clarity on the purpose of the portal and limited awareness. Regarding the purpose of the portal, some participants questioned why the portal was implemented, “why do I need to use it? What’s it for?” (P018, female, 70 years, Cantonese speaking). It was further reported that the portal appeared to have limited value as its functions were very limited:

*by just receiving a text message to say yes or no, you know, you can get very, like cheap solutions to do that*.

[P007, female, 54 years]

Similarly, participants explained that they were not aware of all of the portal’s capabilities, such as the functionality to view past appointments and send messages to the clinic. Other participants incorrectly said that the portal could only be used to send messages to the clinic:

*Like it was good that you could leave a note and leave a message. But apart from that, there’s nothing else there*.

[P015, female, 42 years]

It was revealed that certain staff members had limited awareness of the portal. Specifically, participants noted that staff in charge of booking appointments were unaware that patients would receive a text message from Florence once appointments were booked:

*I made an appointment and I said so I’ll be getting a message from, with Florence, and she’s like we don’t use Florence. ... And then as soon as I hung up from her, I got a text message from Florence.*

[P007, female, 54 years]

### Patient’s Recommendations for Improving the Portal

Some participants described improvements they would like to see in the portal. These include translating the portal to other languages to improve inclusivity for CALD people, providing accessibility support/functionalities to improve inclusivity for people with visual or hearing impairments, providing clarity on the send message function and others. A full list of recommendations is listed in Appendix C in Multimedia Appendix 1.

### Scenario-Based Usability Evaluation

In total, 17 patients participated in the scenario-based usability evaluation. There were more females (n=11, 65%) than males (n=6, 35%), and the mean age of participants was 70 (range 62‐84) years. Two participants were eligible by age only, while 15 were eligible by CALD status only. One of the 2 English-speaking participants was 62 years old. Of the 15 CALD participants, 7 spoke Mandarin, 5 Cantonese, 2 Tagalog, and 1 Vietnamese.

All participants were able to log into the portal, but for many (65%), this required multiple attempts, and most (82%) required assistance. Most (94%) participants were able to confirm their appointments, with only 24% requiring assistance. Only 3 participants (18%) were able to successfully send a message to the clinic, despite the majority (n=11, 65%) attempting this task multiple times, as can be found in Table 3.

**Table 3.** Usability test results reporting the effectiveness of completing 7 tasks in the portal.

Tasks	Completed, n (%)	First attempt, n (%)	Multiple attempts, n (%)	Assisted, n (%)
Logging in with OTP^a^	17 (100)	6 (35)	11 (65)	14 (82)
Rescheduling	16 (94)	15 (88)	2 (12)	11 (65)
Confirming	16 (94)	15 (88)	2 (12)	4 (24)
Send message	3 (18)	6 (35)	11 (65)	8 (47)
Log out	10 (59)	10 (59)	7 (41)	5 (29)
Log in second time	12 (71)	11 (65)	2 (12)	5 (29)
Read message	13 (76)	12 (71)	5 (29)	8 (47)

a OTP: one-time password.

Table 4 below shows problem areas identified during usability testing. These problems were mapped to an existing guideline for designing for older adults that was violated in the portal evaluated [16].

Textbox 1 shows the set of recommendations that emerged following review and discussion of the findings, to improve the patient portal design.

**Table 4.** Problem areas identified during usability testing.

Category	Design problem	Tasks	Number of participants observed	Design guidelines for older adults
Inclusivity problems	It was difficult for CALD^a^ users to use the portal without translation assistance	All tasks	5	Familiar language, terms and labels should be used and connections between concepts should be made explicit Use of relevant cues such as pictures can also be helpful, especially for CALD users
Inclusivity problems	There was no function to increase text size or convert speech to text	All tasks	2	To assist the aging eye, 10-point font has been recommended for web pages and/or functionality to adjust the size Having relevant information channels for users, to address individual differences in ability is recommended
Content issues	Purpose of the check-in button was unclear	Rescheduling appointment	1	Clarify the purpose of the tool in a manner that ensures compatibility with a user’s mental model
Content issues	There was no landing page and the purpose of the portal was unclear	Logging in	4	Designs should make use of previous experience as users of new tools will try to make their task manageable by relating what is new to what they already know
Content issues	The purpose of the ’reschedule’ button was confusing as users are only able to request a reschedule rather than actually selecting preferred dates or times	Rescheduling appointment	2	Clarify purpose of functionality in a manner that ensures compatibility with a user’s mental model
Display issues	The size of the “agree box” was too small and nearly invisible	Logging in	8	Choose input and output elements that are easy to perceive and manipulate; for instance, use large fonts, and high contrast displays
Display issues	It was difficult to differentiate between buttons and labels, eg, unconfirmed and confirmed appointments	Confirming appointment	3	Choose input and output elements that are easy to perceive and manipulate; for instance, high contrast displays, and adequate spacing of selectable elements
Display issues	The “new message” button was confusing as new could mean a user has a new message whereas it currently means the user can write a new message	Sending a message to the clinic	1	Clarify the purpose of functionality in a manner that ensures compatibility with a user’s mental model
Display issues	It was hard to identify new messages from the clinic as it is only a short subject line in bold	Reading a message from the clinic	6	Signal different modes for an interface by using strong cues such as color, location, and size
Display issues	The disclaimer message on the “new message” page was too large, covered half the page, and detracted from the purpose of the page	Sending a message to the clinic	4	Minimize scrolling operations
Layout concerns	It was hard to locate the “send message” function	Sending a message to the clinic	9	Organize menus and websites so that the most frequently used information is at the top of the hierarchy Minimize the number of steps in a procedure
Layout concerns	It was hard to locate the “log out” function	Logging out	5	Clarify purpose of functionality in a manner that ensures compatibility with a user’s mental model
Limited functionality	Users could confirm an appointment that has been flagged for rescheduling, which could result in an appointment a patient inadvertently failed to attend	Confirming appointments	1	Avoid errors by designing them out, for instance, by conducting error-checking for faulty user inputs
Poor navigation	Hard to navigate between browser and text message to fetch the OTP^b^	Logging in	14	Provide relevant support by placing information into the world (display) rather than forcing users to keep it in mind Minimize the number of steps in a procedure Avoid or provide alternatives for difficult-to-perform gestures such as pinching, zooming, and multi-finger swiping actions
Poor navigation	The size of the clickable area for new messages was too small, resulting in users clicking on whitespace or timestamps	Sending messages to the clinic	1	Choose input and output elements that are easy to perceive and manipulate; for instance, use large fonts, high contrast displays, and adequate spacing of selectable elements

a CALD: culturally and linguistically diverse.

b OTP: one-time password.

Textbox 1. **Design recommendations to improve the portal.**

- Increase the size of the “agree” box and improve the visibility of the box by using color, contrast, and outline
- Create a landing page when patients log in to the portal, which provides information on its purpose and functions
- Consider changing the location of the “send message” function to one of the visible buttons on the appointments page
- Consider including the universal symbol for writing a new message next to the “new message” button and consider including “to” or “from”
- Make new messages from the clinic more obvious to the user using colors or shading, symbols, icons, or text, for example, “NEW”
- Increase the size of the clickable area for new messages
- Reduce the size of, or consider relocating, the disclaimer message on the inbox page to a pop-up box before the message is sent
- Consider changing the location of the “logout” function to the profile page
- Clearly differentiate between the clickable and unclickable elements (eg, unconfirmed vs confirmed appointments) by using color, shading, contrast, and outline
- Remove one link from the SMS sent to the patients to access the portal
- Provide the option of selecting the preferred language, consider how this impacts messages sent to and from the clinic via the portal
- Consider including accessibility options, for example, for users to dictate their message in their preferred language rather than type it, and to increase the font size
- Consider removing the “check in” button if not functioning at the clinic
- Gray out or disable the “confirm” function once a patient sends a request to reschedule an appointment

## Discussion

### Principal Findings

In this study, we found that the usability of a patient portal and the approach to its implementation in a real-world setting could be improved, particularly for CALD and older people. In terms of system usability scores, older adults recorded significantly lower scores, although CALD people did not. Nonetheless, in the qualitative interviews, CALD participants reported language as a factor contributing to poor usability and the need for translation assistance for CALD people was noted in the usability testing, which represented a barrier to uptake. Participants noted a range of benefits of using the portal, specifically relating to the ease of managing their appointments. Ease of use and the simplicity of the portal were key factors commonly reported among younger users. However, design problems, including inadequate incorporation of features that maximize inclusivity, were found to limit the usability of the portal for some participants in our study. Also, we found that the purpose of the portal was unclear to some patients and had no impact on the ease of changing appointments. In addition, some relevant staff members were not aware of, or trained on, the portal and how it might impact their work with patients. Taken together, our study elucidates the importance of careful implementation and inclusive design approaches to ensure usability and engagement with patient portals in real-world settings.

Seminal works have established a range of models (eg, Inclusive Design Model) [32], guidelines (eg, Web Content Accessibility Guidelines) [16,33], toolkits (eg, Inclusive Design Toolkit) [34], and key user capabilities (eg, vision, hearing, thinking, mobility, reach, and dexterity) [35] that must be considered to produce an inclusive design. A design is inclusive when it is produced through thorough consideration of the skills and capabilities of the end-users [36]. However, our study revealed that the portal design was not fully aligned with existing inclusive design guidelines, which is likely to result in problems for many groups of users. For example, some participants reported that the “agree box” was too small, a particular concern for people with visual impairment. Others described challenges with using the OTP, a concern that may adversely affect people with memory limitations. And some participants reported on their inability to use the portal due to language barriers, implying limited consideration of people who identify as CALD. These findings add to existing literature suggesting that inclusive design of patient portals is not yet normalized [37-39]. We therefore call for better adherence to existing design guidelines for older adults when designing patient portals, such as the ones identified as violated in this study (Table 4 [16]).

Despite an increased number of usability evaluation studies on patient portals being published in the past few decades, specific design guidance on how to improve inclusivity, particularly for CALD and older people, has not had corresponding growth [12]. In a review of 85 articles on patient portals on usage behavior and usability, 29 studies reported on usability, with the majority (18, 62% studies) using self-report surveys [40]. Another review of 120 articles on patient portals identified 20 usability studies and revealed that the majority (14, 70% studies) assessed usability with subjective or self-report methods (eg, surveys and/or interviews) [13]. The authors found that in-depth objective usability studies were more effective at uncovering a variety of design problems but were rarely used [13]. Whilst useful, survey methods only report on whether there are usability concerns, and which groups are most impacted, rather than the specific design problems. In our study, the PREMs and postimplementation survey results indicated that there were usability problems, but the combination of interviews and objective usability evaluation uncovered the specific design problems contributing to poor usability. Therefore, we stress the importance of incorporating different perspectives, as was done in our study, to better identify design problems and provide guidance on how to improve usability.

Our study also revealed that the purpose of, and the functionalities in, the portal were unclear to some patients, preventing uptake. This indicates some potential gaps in the implementation approach. A systematic review of 18 studies on interventions to improve the uptake of patient portals in vulnerable populations found that most interventions were focused on the individual, rather than the organization [23]. To increase portal uptake, the authors further identified organization-based strategies such as the use of various promotion and education techniques to increase patients’ exposure to the portal, aligning with our research findings [23]. Therefore, we recommend that health care organizations aiming to implement patient portals should use a suite of pre- and postimplementation strategies to better engage patients and to facilitate uptake.

Based on the findings of this study, we provide a range of broad recommendations on patient portals for health service organizations and designers. Health service organizations should engage with patients early on in the process of co-designing the portal; carefully target under-served populations for early feedback; craft strategies to create awareness before and after roll-out of the technology; and train staff members whose roles and responsibilities may be impacted by the implementation of the portal. Furthermore, designers should use familiar language, terms, and cues to ensure inclusion of different patient demographics; minimize the number of tasks and actions users need to remember and act on; carefully design accessibility functionalities to cater to the needs of older adults; and ensure clarity of the portal’s purpose.

### Strengths and Limitations of the Study

To our knowledge, this was the first study to use a mixed methods approach, including pre-post PREMs evaluation, usability survey, semistructured interviews, and scenario-based usability testing to evaluate patients’ experience of using a web portal in a dental outpatient setting, particularly from the perspective of older adults and those who identify as CALD. Our approach allowed us to identify design problems and how these may be optimized in future portal designs. Limitations of our study include that data were collected in a single health care organization, which may limit the generalizability of our results to dissimilar organizations. Also, as the pre-post PREMs evaluations were anonymous, we were unable to match the data between pre- and postimplementation patients, and the sample may have been different, although Table 1 indicates good balance between the demographic characteristics of the pre- and postimplementation samples. We also could not track participant involvement across all the data collection methods used due to ethical restrictions. Although self-report methods (eg, interviews and surveys) were used, which are prone to bias, our scenario-based usability testing was based on participant observations. Our study revealed emergent findings for the CALD group, but we did not include a comparator group in our usability testing, so we recommend further evaluation of patient portals for this subgroup beyond language barriers. Future work will involve evaluating the impact of our design recommendations to determine the extent to which they enhance the usability of the portal.

### Conclusion

A benefit of patient portals is their potential to improve efficiency in the use of health service resources in outpatient settings, but this can only be realized if the portal is usable and used. Our study found no impact of the portal on the ease of changing appointments and revealed areas where the usability of the patient portal and the approach to its implementation could be improved. There is a need to better leverage inclusive design principles when developing patient portals, and objective usability evaluation methods when assessing the portals to ensure they are usable and can be used by all patients.

## Supplementary material

10.2196/74275 Multimedia Appendix 1 Additional files.

## Abbreviations

CALD: culturally and linguistically diverse
FTA: failure to attend
OTP: one-time password
PREM: patient-reported experience measure
SUS: Simplified System Usability Scale
