# Supplementary material for: Toward Normalizing Inclusive Design by Uncovering Patient Experiences of a Web Portal in a Dental Hospital: Mixed Methods Study
Source: J Med Internet Res. 2025 Aug 14;27:e74275. doi: 10.2196/74275 (PMC12352587; doi:10.2196/74275)
Supplement: Multimedia Appendix 1 [file jmir-v27-e74275-s001.docx]

**Supplementary Files**

**Appendix A - PATIENT INTERVIEW GUIDE**

**Introduction**

Hello, my name is [ENTER NAME]. I am a research fellow from the University of Sydney and we are working with the [xxx] Dental Hospital on this research project.

The purpose of this interview is to explore people’s experiences of using Florence. We would like to know your thoughts about the tool.

After the interview, we will transcribe the audio recording and remove any identifying information. Only researchers involved in the study will have access to your responses and there will be no information that can identify you.

Do you have any questions for me?

**Tool use**

1. Did you use Florence at [xxx] Dental Hospital’s [de-identified] Clinic?

**User satisfaction**

2. What is your overall opinion of Florence? (likes and dislikes)

- What did you like about Florence
- What did you not like about Florence

3. How do you think Florence is helpful? What are the main benefits of Florence?

- What components/features are useful?
- Is it particularly useful for some things?
- Was it able to make appointments easy to manage

4. Was Florence easy to learn and to use?

- Can you describe any problems you had with Florence?
- Did you have to ask for help from a family member or friend to use it?

5. How can we improve Florence?

- What additional functions or features will be useful to have?
- Should it be available in different languages?

6. How do you feel about rolling out Florence to all patients in [the] Health Service?

**Demographics**

6. What gender do you identify as?

7. What is your age?

8. What language do you speak most at home?

9. How comfortable are you with using technology?

1. Do you own a computer or laptop or iPad?
2. Do you own a smartphone (e.g. iPhone)?
3. Do you use a computer or smartphone regularly?
4. (Ask if answer is ‘no’ to a, b, and c) Do you have access to a computer or smart phone through family, a friend, at a place or work etc.?

**Appendix B - Florence usability test instructions**

*Thank you for participating in our study aimed at testing how well Florence works for you. There are five main tasks we will like you to please complete. Please let us know what pops into your head as you do the tasks.*

| **TASK 1: Logging in using the one-time password (OTP) code** |
| --- |

*You have scheduled an appointment at the [xxx] Dental Hospital. You will now receive a text message (SMS) on your mobile phone notifying you that you have an appointment.*

- *Please log in using the one-time password code*

| **TASK 2: Rescheduling appointment** |
| --- |

*You are now on your profile page. You have an upcoming appointment. You suddenly realise you have a planned lunch appointment with a friend visiting from overseas on the same day you have your dental appointment. You would like to meet your friend for the lunch appointment and reschedule your dental appointment.*

- *Please use Florence to request a re-schedule of your dental appointment*

| **TASK 3: Confirming appointment** |
| --- |

*You have a second upcoming appointment that you would like to confirm.*

- *Please use Florence to confirm that you will attend the upcoming appointment*

| **TASKS 4 and 5: Sending and a message from the clinic** |
| --- |

*You have confirmed the upcoming appointment but you don’t know how to locate the clinic at [xxx] Dental Hospital.*

- *Please use Florence to send a message to the clinic and ask for the location*
- *Log out of Florence*

| **TASKS 6 and 7: Logging in and reading a message from the clinic** |
| --- |

*You have sent a message to the clinic asking for the location and the clinic has now responded to you.*

- *Login and read the message*

***Thank you so much for participating in this study. Feel free to have a chat to our moderator about anything else you would like us to know about Florence.***

**Appendix C:** List of improvements suggested by patients

| **Improvement** | **Quotes** |
| --- | --- |
| Translate the portal to other languages to improve inclusivity for CALD people | “*Sometimes hard for me too because my English is not very good…Mandarin reading is best for me…Like I fully understand yeah, if Mandarin, yeah*.” – P004.  “*saw a lot of Vietnamese and Chinese people there. And a lot of Greek people as well…And like they were from different nationalities that I saw when I was there that had relatives with them, and they had to translate on their behalf*.” – P015 |
| Provide accessibility support/functionalities to improve inclusivity for people with visual or hearing impairments | “*they might have a phone but they don’t get their eyes checked, and they can’t read or see*” – P017.  “*a talk feature possibly for somebody that is impaired…some people just rely only on a guide dog. So, you know, if there was a feature for a blind impaired person, possibly that could be a bonus*” – P002 |
| Provide clarity on message sending function | “*even the way it's designed, it doesn't really tell me that it goes straight to the clinic… So if I want to message, I don't know where that message is going. Is anyone going to receive it or am I gonna get a response?*” – P007 |
| Include function to add appointments to phone calendar | “*Just maybe an autolink to the calendar, like giving you that option where it's like, you know, do you want us to save this to your calendar*” – P015 |
| Provide address of the clinic for each appointment | “*It never had full addresses…Maybe just put a full address down and maybe even a contact number*” – P011 |
| Consider delivering portal via an app | “*I would actually prefer my own my application, where I know these are all my all my appointments, that I can just click on to at any given time”* – P007,  “*if it was an app, I could just put it on my phone leave it there, you know, go in and out as I saw fit. But now I've just got to keep a message there to go in and check it*.” – P010 |
| Provide ability to reschedule appointments via the portal | “*definitely the rescheduling. Definitely that because you cannot do that on the thing. So you know, that would, that would be a major like improvement because that would really annoyed me like, oh, gosh, I've got to call them again*.” – P012.  “*seeing if, like, if there's other times available within that date, you can just see it as an option*” – P015 |
